# ^14^N overtone NMR under MAS: signal enhancement using symmetry-based sequences and novel simulation strategies†
†Electronic supplementary information (ESI) available. See DOI: 10.1039/c4cp03994g



**DOI:** 10.1039/c4cp03994g

**Published:** 2015-02-09

**Authors:** Ibraheem M. Haies, James A. Jarvis, Harry Bentley, Ivo Heinmaa, Ilya Kuprov, Philip T. F. Williamson, Marina Carravetta

**Affiliations:** a School of Chemistry , University of Southampton , SO17 1BJ , Southampton , UK . Email: m.carravetta@soton.ac.uk; b Department of Chemistry , College of Science , University of Mosul , Mosul , Iraq; c School of Biological Sciences , University of Southampton , SO17 1BJ , Southampton , UK; d National Institute of Chemical Physics and Biophysics , Akadeemia tee 23 , Tallinn , 12618 , Estonia

## Abstract

Overtone ^14^N NMR spectroscopy is a promising route for the direct detection of ^14^N signals with good spectral resolution. Its application is currently limited, however, by the absence of efficient polarization techniques for overtone signal enhancement and the lack of efficient numerical simulation techniques to aid in both the development of new methods and the analysis and interpretation of experimental data. In this paper we report a novel method for the transfer of polarization from ^1^H to the ^14^N overtone using symmetry-based R-sequences that overcome many of the limitations of adiabatic approaches that have worked successfully on static samples. Refinement of these sequences and the analysis of the resulting spectra have been facilitated through the development of an efficient simulation strategy for ^14^N overtone NMR spectroscopy of spinning samples, using effective Hamiltonians on top of Floquet and Fokker–Planck equations.

## Abstract 2

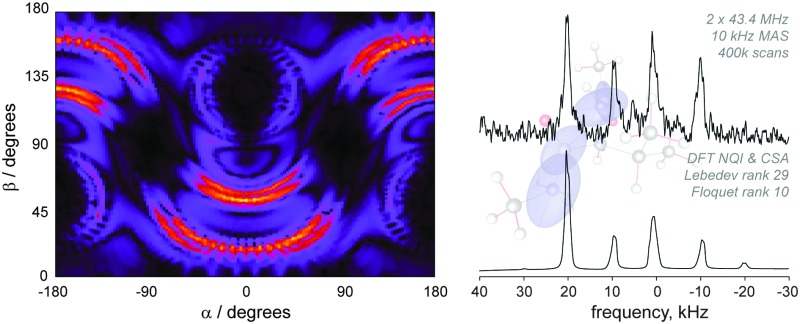
Overtone ^14^N NMR spectroscopy is a promising route for the direct detection of ^14^N signals with good spectral resolution.

## Introduction

Nitrogen is a key element in both natural and synthetic systems, present in the amino acids and nucleotides that constitute the building blocks of biological systems as well as in many man-made materials such as polymers and pharmaceuticals. Accordingly, NMR methods for the analysis and characterization of the nitrogen sites within these materials are of great interest. Nitrogen has two naturally occurring NMR active isotopes: ^15^N with nuclear spin 1/2 and a natural abundance of 0.37%, and ^14^N with nuclear spin 1 and a natural abundance of 99.6%. The majority of nitrogen NMR studies to date have used ^15^N because of the intrinsically sharper lines and ease of manipulation, albeit frequently at the expense of isotope labeling due to its low natural abundance. Despite its high natural abundance, ^14^N NMR studies are frequently complicated by the large quadrupolar interaction. In contrast to half-integer spin quadrupolar nuclei, where the central transition is located close to the Larmor frequency and unaffected by the quadrupolar interaction to first order,^1,2^ the single-quantum transitions of ^14^N are frequently distributed over a MHz frequency range and distant from the Larmor frequency, complicating the detection of the NMR signal.^3–5^ The large quadrupolar interactions also limit the application of magic-angle spinning, a technique designed to improve resolution and sensitivity for powder samples through the averaging of anisotropic interactions.^3^ Accordingly, most ^14^N investigations have focused on single crystal studies or nitrogen sites that exhibit a small (<100's kHz) quadrupolar coupling, such as those with high symmetry,^3,6–10^ while for large quadrupolar interactions (>1 MHz) step-wise acquisition can faithfully reproduce the quadrupolar line shape.^3,11,12^ In recent years, several methods for indirect detection of ^14^N under MAS have been developed, where the ^14^N signal is characterized indirectly through its interaction with a nearby spin-1/2 nucleus (typically ^1^H or ^13^C),^5,13–24^ but a high-resolution method for direct detection of ^14^N is currently lacking.

An alternative approach to ^14^N NMR relies on the detection of the so-called overtone transition which corresponds to the change in spin projection quantum number by Δ*m* = 2, formally a double-quantum transition, with direct detection at twice the Larmor frequency.^25^ Multiple-quantum transitions are in general forbidden, but the presence of a strong quadrupolar interaction mixes Zeeman energy levels which in turn leads to a non-zero probability for the overtone transition.^26–28^ Overtone transitions are insensitive to the first order quadrupolar interaction and have been observed on both static and spinning samples containing ^14^N since the 1980's.^26–28^ Both direct excitation and cross-polarization excitation schemes have been employed, the latter giving enhancements in sensitivity of up to a factor of 5.9 in single crystals.^27^ O'Dell and co-workers demonstrated that direct excitation of ^14^N overtone transitions with high resolution is possible under MAS,^4,29^ proving that good resolution is indeed achievable in ^14^N NMR. While direct excitation schemes are typically narrow-band, broadband excitation of the ^14^N overtone transition has been achieved using WURST-based methods,^29^ but this leads to a further loss in signal intensity. More recently, very fast MAS experiments on ^14^N overtone demonstrated that the overtone signal can be acquired in the indirect dimension of a 2D experiment using an approach that resembles indirect detection ^14^N NMR experiments.^30–32^ Adiabatic CP methods demonstrated for static overtone excitation have not been successfully extended to rotating solids so far. The only successful report to date of ^14^N overtone based CP methods on rotating solids is from Rossini *et al.*,^30^ who used cryogenic MAS dynamic nuclear polarization (DNP) conditions, where ramped CP was applied to transfer polarization to ^14^N overtone from hyperpolarized ^1^H nuclei. The ^14^N overtone signal is significant,^30^ but the same experiment produced no visible signal in the absence of ^1^H hyperpolarization.

In part the slow development of ^14^N-overtone NMR can be attributed to the absence of efficient simulation strategies, which have been employed so successfully elsewhere for the development of solid-state NMR experiments. Simulation of the overtone transitions is demanding because: (i) there is no rotating frame that would remove all time dependence from the Hamiltonian; (ii) overtone transition moment is small, therefore all ^14^N overtone pulses are very long and cannot be treated as ideal; (iii) the system is time-dependent at both twice the Larmor frequency and at the MAS frequency, and those frequencies are orders of magnitude away from each other; (iv) for powdered samples, three-angle spherical averaging is needed. Currently the simulations are performed by brute force in the laboratory frame and are therefore very time consuming.^4^

In this work we present a new approach based on the PRESTO (Phase-shifted Recoupling Effects a Smooth Transfer of Order), a symmetry based R-sequence, to establish ^1^H to ^14^N overtone correlation and achieve polarization transfer to enhance the ^14^N overtone transition under MAS.^33–35^ Complementing earlier indirect methods that employ high spinning speeds (>50 kHz) and high RF field (>250 kHz), the PRESTO sequence provides significant enhancements at moderate spinning speeds (<20 kHz) and moderate RF fields (<70 kHz), which makes it applicable to larger samples. Through the development of computationally efficient methods for the simulation of ^14^N OT spectra we have been able to simulate the line shapes obtained under both direct excitation and PRESTO, accounting for both finite pulse effects and the polarization transfer process. The algorithm uses effective Hamiltonians on top of Floquet and Fokker–Planck equations. All methods described below are implemented into versions 1.5 and later of the Spinach library.^36^

The PRESTO approach is demonstrated on two model samples, glycine and *N*-acetyl-valine (NAV), which represent two different situations in terms of the magnitude of the quadrupolar interaction. Sensitivity increases significantly with PRESTO, with enhancements factors between 2.5 and 3.8 compared to direct excitation, when comparing the experiments by the number of acquisitions. There is no enhancement from the polarization transfer alone if the signal is measured per unit time on these samples, as the relaxation time for proton is longer than for ^14^N (from literature). We also demonstrate the application of PRESTO technique to the acquisition of ^1^H–^14^N overtone correlation spectra.

## Materials and methods

### Sample preparation

NMR experiments were performed on glycine (identified as the alpha-glycine polymorph from X-ray diffraction) and *N*-acetyl-valine (NAV), both acquired from Sigma-Aldrich and used without further purification.

### Solid state NMR equipment

NMR measurements at 14.1 T were performed using Agilent DD2 600 MHz spectrometer equipped with a 3.2 mm narrow-bore triple resonance T3 style probe and 3.2 mm zirconium oxide rotors. The resonance frequency of the ^14^N overtone signal at this field is expected to be 86.7448 MHz. All 14.1 T spectra were referenced to this frequency.

NMR measurements at 20.0 T were performed using 850 MHz Bruker spectrometer equipped with a 3.2 mm wide-bore triple resonance probe and 3.2 mm zirconium oxide rotors. At this field the ^14^N overtone transitions are expected to be located at 122.8331 MHz. All 20.0 T spectra were referenced to this frequency.

### Solid-state NMR experiments – direct excitation and calibration

At 14.1 T, the RF power level for the ^14^N overtone was calibrated to 55 kHz on a water sample using the ^17^O NMR signal with a resonance frequency of 81.397 MHz. At 20.0 T, the RF power level for the ^14^N overtone was calibrated to 70 kHz on a water sample using the ^17^O NMR signal with a resonance frequency of 115. 262 MHz.

**Fig. 1:**
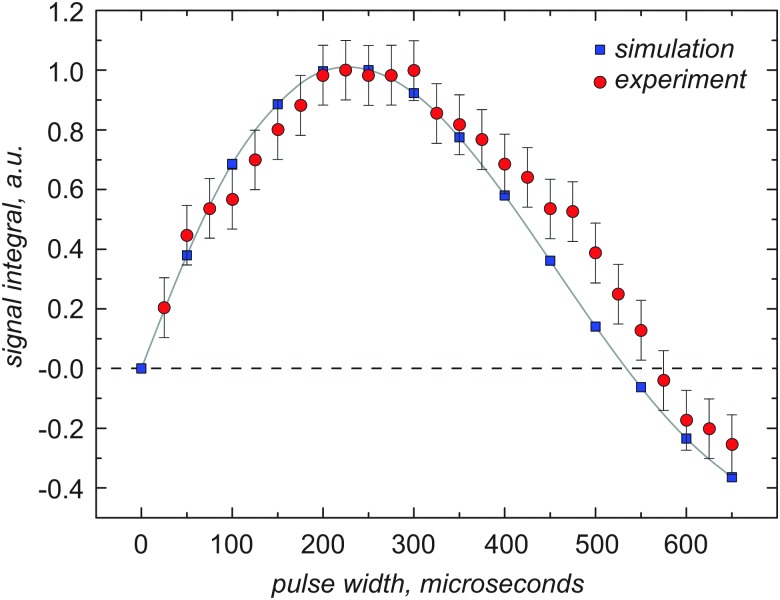
Experimental data and the corresponding simulation of the nutation curve for the ^14^N overtone signal of glycine powder, observed at its +2 spinning sideband under MAS at 9.92 kHz, using an RF pulse with a nominal RF amplitude of 55 kHz, as calibrated on the ^17^O signal from a water sample. The simulation was performed as described in the theory section using experimental parameters for glycine from literature (Table 1), and probe geometry without any fitting or adjustment, except for the arbitrary overall scaling multiplier. The π/2 and π pulses for the ^14^N overtone are found to be 260 μs and 520 μs, respectively.

All experiments were performed with the RF carrier frequency in resonance with the +2 overtone spinning sideband. For direct excitation, the optimal excitation pulse width was found by taking the time of maximum signal intensity with respect to the pulse duration using direct overtone excitation under MAS with a pulse acquire sequence (shown in Fig. 1). Optimal pulse durations for direct detection were found to be 260 μs for both glycine and NAV at 14.1 T and 275 μs for glycine at 20 T.

During acquisition, 72 kHz SPINAL proton decoupling^37^ was applied at 14.1 T and 89 kHz SPINAL decoupling was used for the experiments at 20.0 T. The effect of decoupling during the pulse was considered at 14.1 T and was found to be very minor for glycine, but improved significantly the excitation performance of the NAV signal. The pulse repetition delay for direct overtone excitation and spin echo were set to 0.5 s for glycine and 0.4 s for NAV (these values are compatible with previous overtone studies^4,29^). Repetition delays for PRESTO were set to 2.5 s for both glycine and NAV. Due to probe ringing near the overtone frequency, the first 70 μs of the FID were removed by applying the appropriate left shift of the experimental data points followed by phase correction for all direct excitation experiments.

Data were referenced indirectly to 0 ppm (liquid ammonia) using the ^14^N signal of ammonium chloride at 39.3 ppm.^38^ The overtone reference frequency was taken to be twice the ^14^N reference frequency.

### Solid-state NMR experiments and spin-echo

**Fig. 2:**
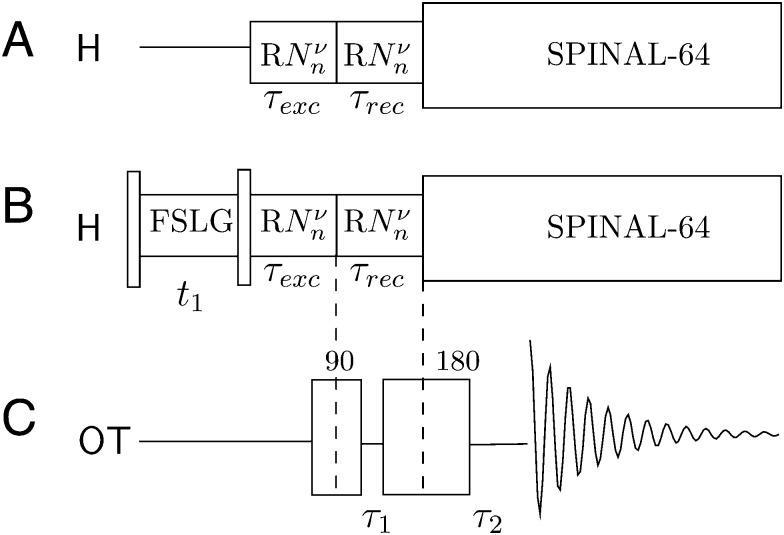
(A) PRESTO-II sequence for polarization transfer from ^1^H to ^14^N overtone, (B) 2D PRESTO, providing ^1^H–^14^N overtone correlation information with homonuclear decoupling (FSLG) during *t*_1_. The two experiments share the same ^14^N overtone channel event sequence, which is an overtone spin-echo sequence (C).

Spin echo experiments were performed at 14.1 T using the pulse sequence in Fig. 2C. Optimal pulse durations are different from the optimal pulse width obtained from direct excitation, but they are equal to the durations used in the PRESTO-II sequence. The π/2 and π ^14^N overtone pulse lengths were set to 360 μs and 720 μs respectively for glycine, 170 μs and 340 μs respectively for NAV. The spin echo experiments used *τ*_1_ = 0 μs and *τ*_2_ = 10 μs for both NAV and glycine. Additional experimental data with longer *τ*_1_ and *τ*_2_ values are presented in the ESI.† The *τ*_2_ delay includes the probe dead-time.

### Solid-state NMR experiments and PRESTO-II

PRESTO-II sequence is described in detail elsewhere^33,34^ and is used here to achieve polarization transfer between ^1^H and the ^14^N overtone transition using symmetry-based *RN* *ν* *n* sequences^35^ that reintroduce the ^1^H–^14^N overtone dipolar interaction under MAS. The variants of PRESTO used in this work are summarized in Fig. 2. The basic principle behind the *RN* *ν* *n* sequences is that only selected NMR interactions can be recoupled to first order, while removing all others, by rotor-synchronizing the sequence in such a way that *NR* pulses (where each *R* pulse is a simple or composite π pulse) fit exactly in *n* rotor periods. The phase of each *R* pulse is determined by the third number *ν* in such a way that *φ*_*R*_ = (π*ν*)/*N* and it is alternated between +*φ*_*R*_ and *–φ*_*R*_. The result is an average Hamiltonian such that:1
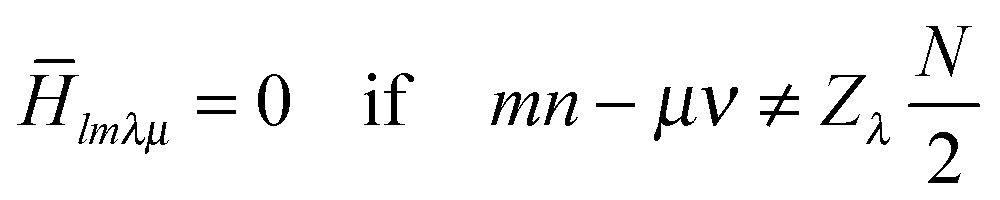
 Different NMR interactions are classified in terms of their space and spin ranks, where *l* is the space rank of the interaction tensor, *m* is the space component, *λ* is the spin rank, *μ* is the spin component and *Z*_*λ*_ is an integer with the same parity as *λ*. For the *R*1852 and *R*1871 symmetries used here, the terms which are recoupled correspond to {*l*,*m*,*λ*,*μ*} = {2,±2,1,±1}, that is, CSA and heteronuclear dipole–dipole interactions.^33–35^ These symmetries were selected based on the practical concerns of available RF power and spinning frequencies in our probe.

The experimental optimization of PRESTO was done in stages. First, the signal intensity was optimized by direct ^14^N overtone detection (described above), then the conditions for the spin echo were optimized. These parameters were used to set up the experiment using PRESTO-II. Then both the π/2 and π ^14^N overtone pulse lengths were optimized again and the best pulse durations were found to be 360 μs and 720 μs respectively for glycine at 14.1 T, 375 μs and 750 μs respectively for glycine at 20.0 T, 170 μs and 340 μs respectively for NAV. Excitation and reconversion times were kept equal and the optimal duration was found to be 231 μs at 20.0 T and 201 μs at 14.1 T. Optimal spin echo pulse durations within PRESTO-II are not necessarily the same, but were found to be similar to the basic spin echo sequence.

PRESTO-II measurements were conducted using the following experimental conditions: spinning frequency *ω*_*r*_/2π = 9.92 kHz for *R*1871 (data shown only as 2D) and *ω*_*r*_/2π = 19.84 kHz for *R*1852. During PRESTO, the basic element *R* = {180_+*φ*_*R*__180_–*φ*_*R*__}_0_ was employed during the excitation block, and *R* = {180_+*φ*_*R*__180_–*φ*_*R*__}_90_ during the reconversion block.

The phase of the excitation block was cycled in two steps to remove contributions coming from direct overtone excitation, both in the experiments and in the simulations. The signal contributions from excitation through the spin echo as well as PRESTO could both be retained without this phase cycling step. For PRESTO-II, a pre-acquisition delay of 10 μs prior to detection was found to be sufficient, as for the overtone spin echo experiments.

Proton RF amplitudes of 89.2 was utilized during PRESTO. For the two-dimensional version of the PRESTO-II sequence displayed in Fig. 2B, Frequency-Switched Lee–Goldburg (FSLG) decoupling was used during the *t*_1_ time interval to suppress the homonuclear ^1^H couplings and improve resolution. The proton RF amplitude was set to 89.2 kHz corresponding to a LG frequency offset of 63 kHz.^39^

The narrow bandwidth of the PRESTO experiments is attributed to the overtone spin echo step in the pulse sequence, which is currently using simple sinusoidal wave ^14^N overtone pulses.

### Theoretical methods

Overtone MAS NMR is a difficult simulation target. The primary reason is the presence of two distinct large frequencies in the laboratory frame spin Hamiltonian:^40^
2
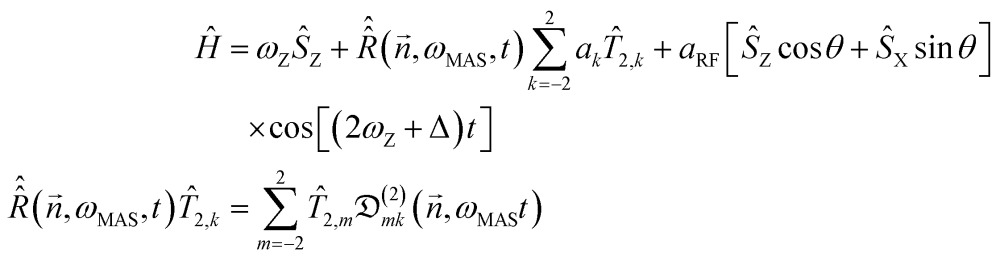
where *ω*_Z_ is the Zeeman precession frequency, 
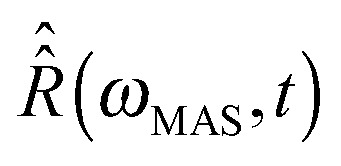
 is a spinning superoperator, *n* is the spinning axis, *ω*_MAS_ is the spinning rate in angular frequency units, {*a*_*k*_} are the five irreducible spherical components of the quadrupolar interaction tensor, *T*_2,*k*_ are the corresponding irreducible spherical tensor operators,^41^ *𝔇*(2)*mk*(*n*,*φ*) are second-rank Wigner *D*-functions in angle-axis parameterization,^42,43^ *a*_RF_ is the radiofrequency (RF) irradiation amplitude, often called the RF nutation frequency (in some conventions requiring a factor of 2 in front^34^), *θ* is the angle accounting for the coil orientation relative to the magnet and Δ is the offset of the RF irradiation frequency relative to the frequency of the double-quantum transition. From the computational perspective the following complications arise:

(1) There is no convenient rotating frame. The standard Zeeman rotating frame normally used in high-field MAS NMR^44,45^ is ineffectual against the double-quantum frequency under the cosine. A double-quantum rotating frame would flip the sign of the Zeeman term, but not eliminate it. Laboratory frame simulations require two points per period of the fastest oscillation, meaning many millions of propagation steps per evolution second in a 14.1 T magnet, taking hours to days on modern workstations.^4^

(2) The pulses cannot be assumed to be ideal (defined as “short enough for the background Hamiltonian evolution to be negligible during the pulse”) – the mixing caused by the strong quadrupolar interaction means that the transition between the outer energy levels under *ŝ*_X_ is no longer forbidden,^46^ but the transition moment for the overtone is two orders of magnitude smaller than that of the fundamental frequency. At the limit of the available hardware, the 90 degrees pulse on ^14^N overtone is around 200 μs long. The RF pulse term in eqn (2) cannot be avoided by starting the simulation from *ρ̂*_0_ = *ŝ*_X_ as the initial condition and a finite-duration pulse must be properly accounted for.^29^

(3) The system is spinning with a frequency typically in the range of 10–70 kHz, which is three orders of magnitude away from the frequencies of the spin evolution processes. This makes the Hamiltonian time-dependent in a numerically difficult way. At the matrix exponentiation stage, the difference between the adjacent time slices of the laboratory frame propagation problem are perilously close to the numerical accuracy limits of double-precision arithmetic.

(4) The system is a powder and a spherical average is therefore required. The lab frame simulation with millions of time steps would have to be repeated thousands of times to obtain a three-angle powder pattern.^44,45,47^ Our simulations indicate that the spherical integration grid ranks required to converge an overtone powder pattern are considerably greater than those needed for the single-quantum transition signal with a comparable number of spinning sidebands.

To overcome these issues we have developed the following simulation method that avoids the time-consuming laboratory frame time propagation. Its stage are:

(1) The time dependence is eliminated from the MAS part of eqn (2) by switching it into Floquet^48^ or Fokker–Planck^49^ formalism (both give identical results), in which MAS is treated as a time-independent interaction between spin space and lab space degrees of freedom. In the Fokker–Planck picture^49^ we get:3
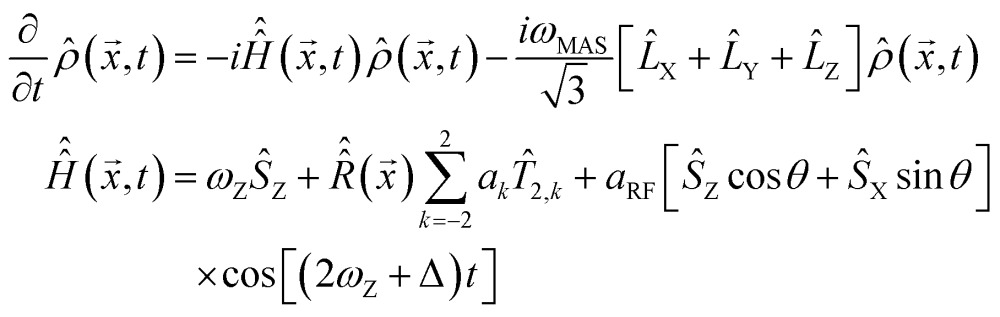
where 
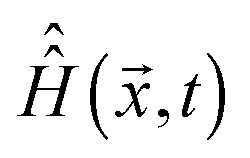
 denotes a Hamiltonian commutation superoperator, 
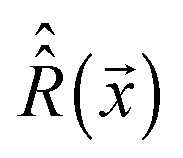
 is now a time-independent rotation into the orientation specified by *x* and {*L*_X_, *L*_Y_, *L*_Z_} are angular momentum (not spin) operators. Spherical averaging within the Fokker–Planck MAS formalism is performed automatically.^49^ The Floquet picture is similar,^48^ but a two-angle spherical integration grid is still required:4
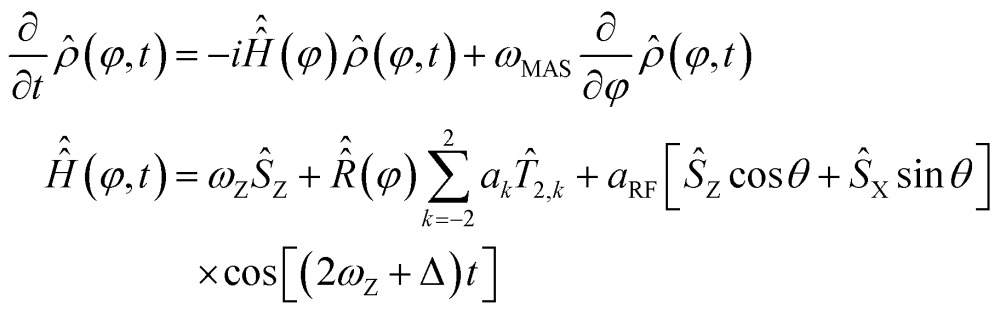
where *φ* is the spinner phase. Both formalisms eliminate explicit time dependence from the MAS part of the problem – the time-dependent Hamiltonian problem is mapped into a time-independent Hamiltonian problem in a space of higher dimension. The increase in the matrix dimension is by a factor of 2*l* + 1 (Floquet) and (1 + *l*)(1 + 2*l*)(3 + 2*l*)/3 (Fokker–Planck), where *l* is the cut-off rank (approximately equal to the number of spinning sidebands expected in the simulation). This dimension increase used to be a significant limitation,^50^ but recent developments in sparse matrix manipulation techniques,^51^ in Krylov subspace techniques^52^ and particularly in tensor train methods^49,53^ have removed the problem – spin Hamiltonian matrices with dimensions in the millions are now handled routinely,^53,54^ although the functionality in question currently appears to be unique to *Spinach* library.^36^ Floquet and Fokker–Planck methods are also preferable to time slicing from the end user perspective – both methods treat MAS as a static interaction within the Hamiltonian, thereby avoiding the complicated event scheduling^55^ during pulse sequence execution and signal detection: pulse sequences programming becomes easy.

(2) The effective Hamiltonian method,^56^ using matrix logarithms, is employed to remove the time dependence from the pulse part of eqn (2). The matrix logarithm technique is used because the corresponding Magnus expansion^57^ does not converge even at the fourth order, due to the above mentioned lack of convenient rotating frame in overtone NMR. We could not find a way around the slow convergence of the Magnus series and the exact effective Hamiltonian expression is therefore used instead:5
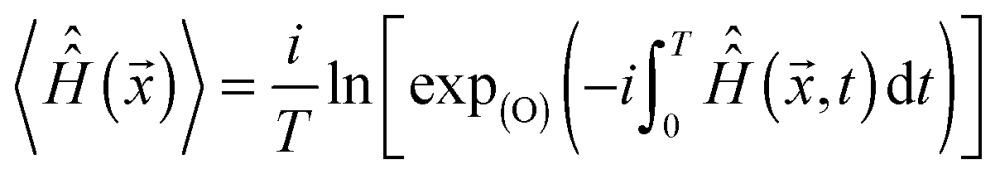
where *T* = 2π/(2*ω*_Z_ + Δ) is the RF period and exp_(O)_ denotes a time-ordered exponential. The latter is computed by slicing the RF period *T* and multiplying up slice propagators – sixteen slices using a second-order product integral quadrature^58^ were in practice found to be sufficient. Matrix logarithm is not a straightforward operation, but numerically stable methods have recently emerged.^59^Eqn (5) eliminates the last time-dependent term without making any assumptions about the RF period or its relation to the MAS period. The Hamiltonian is now time-independent. Importantly, the total cost of getting this far is only a few dozen matrix operations.

(3) Pulses are applied using propagator squaring. The effective Hamiltonian in eqn (5) is over the RF period (about 10 nanoseconds), but the following relation:^55^
6
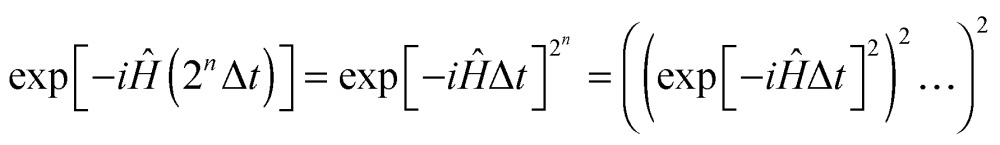
allows to amplify this time step exponentially quickly, by squaring the propagator. A 200 μs pulse is obtained at the cost of about 17 squaring operations. The resulting propagator is then applied to the system in a single multiplication – this highlights one advantage of exponential propagation compared to linear multistep methods.^4^ This final operation takes the problem to the signal detection stage, where the system evolves freely from the state |*ρ̂*_0_ that it has reached at the end of the pulse.

(4) The problem is moved into the frequency domain before the detection stage simulation is run. The overtone spectrum only occupies a few hundred points within a very large laboratory frame frequency range – it is therefore advantageous to replace millions of points that would be needed in the time domain with a few hundred points directly evaluated in the frequency domain. With a known initial condition and a time-independent Hamiltonian (both of which are available as a result of applying stages 1–3) the problem can be moved into the frequency domain analytically:7
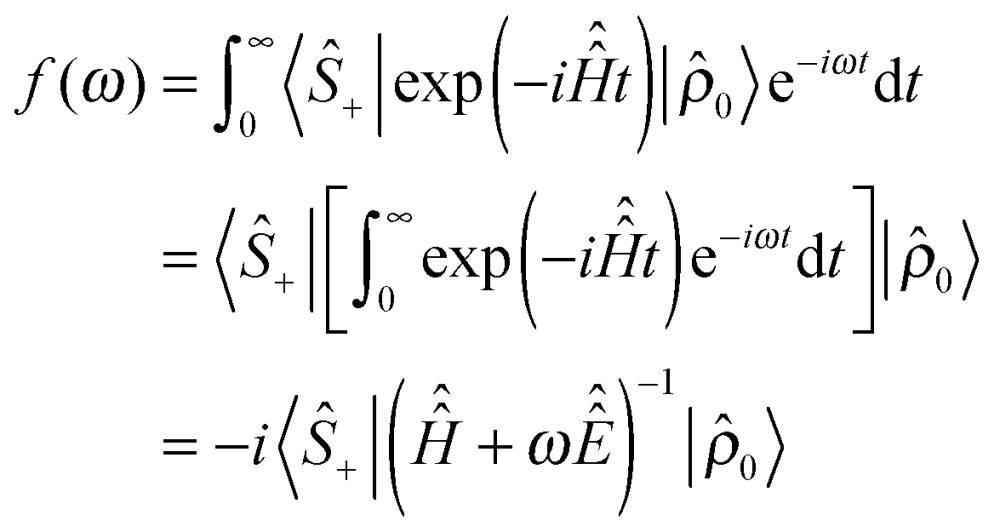
where *ŝ*_+_ is the quadrature detection state, 
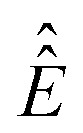
 is the unit operator of the same dimension as 
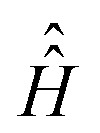
 and the Hamiltonian itself no longer contains the pulse term:8
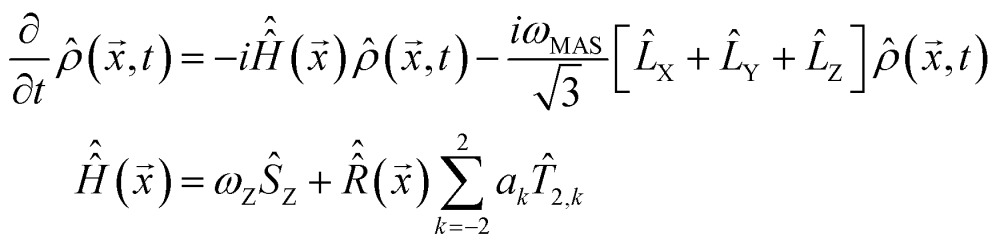
 In practical calculations it is important to make sure that 
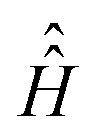
 contains a relaxation superoperator to regularize the denominator in eqn (7) – this is the frequency domain equivalent of signal apodization. Another useful practicality is that the matrix–inverse–times–vector operation (known as *backslash* ^60^) is computed directly and much faster than a matrix inverse followed by a matrix–vector multiplication. The individual frequency points also correspond to independent calculations that may be sent to different CPUs of a large parallel computer.

Nuclei other than ^14^N are kept in their corresponding rotating frames for the entirety of the simulation. The simulation procedure described above is implemented in version 1.5 of *Spinach* library^36^ available at ; http://spindynamics.org. Complete annotated source code for the simulations performed in this paper is included with the example set. For the systems described below, the wall clock simulation time depends on the number of spectrum digitization points as well as target accuracy, determined by Wigner *D*-function rank in the Fokker–Planck formalism^49^ and spherical integration grid rank in the Floquet formalism,^48^ but is generally of the order of minutes to hours.

## Results and discussion

### Direct excitation ^14^N overtone experimental spectra and simulations

The ^14^N overtone resonance position depends upon the isotropic second order quadrupolar shift and the chemical shift. Here we have optimized all experiments for the second (+2) spinning sideband, as this is the most intense sideband, which can be seen from the spectrum acquired for glycine (ESI,† Fig. S1 and previous studies^4,29^). To obtain an efficient spin echo and validate the simulation strategies employed to simulate the effects of the weak pulses on exciting the overtone transition, nutation curves for the ^14^N overtone signal under MAS were measured at 14.1 T at a RF amplitude of 55 kHz (Fig. 1). For glycine at 9.92 kHz the agreement between experimental data and simulations is excellent, using the experimental parameters summarized in Table 1 for glycine and appropriate for the probe geometry, and demonstrate that the ^14^N overtone finite-duration pulses are accurately simulated. The excitation pulses are significantly longer than would be predicted based on nutation curves measured on model compounds, in this case the ^17^O signal of water, because we are dealing with a partially forbidden transition. For spin echo experiments and PRESTO, optimal pulse duration differs from the apparent 90 degree pulse and was found to be 360 μs.

**Table 1:** Summary of parameters used for the simulations for glycine and NAV. The motional averaging of the NH_3_ rotation is accounted for by including a single H–N dipolar interaction along the rotation axis, with an inter-nuclear distance of 1.28 Å. In both cases Floquet theory convergence is achieved at rank 5

	*C*_Q_ (MHz)	*η*_Q_	*r*_NH_ (Å)	Euler angles, DD^*a*^	*σ*_ISO_ (ppm)	Δ*σ* (ppm)	*η*	Euler angles, CSA^*a*^	Lebedev grid rank
Gly^9,63^	1.18	0.53	1.28	[0, 0, 0]	32.4	—	—	—	77
NAV^64–66^	3.21	0.32	1.06	[0, 90°, 0]	121	105	0.23	[–90°, –90°, –17°]	131

^*a*^Dipolar and CSA interaction tensor orientations are quoted relative to the eigenframe of the ^14^N quadrupolar interaction tensor.

For NAV, the signal we observe is very weak, possibly also because of the long dead time of 70 μs. Even though simulations suggest shorter pulses are needed for systems with large quadrupolar interaction,^32^ a reliable nutation curve as in Fig. 1 was not obtained for NAV as for long pulse widths we burn a hole through the overtone line shape. The NAV signal shows a local maximum near 75 μs but after a small drop it increases again, and the “global” maximum signal intensity is observed close to 260 μs, and therefore this value was used for the direct excitation measurements reported here. The optimal pulse duration for echo measurements on NAV was found to be 170 μs. These cannot be related to well-defined flip angles.

**Fig. 3:**
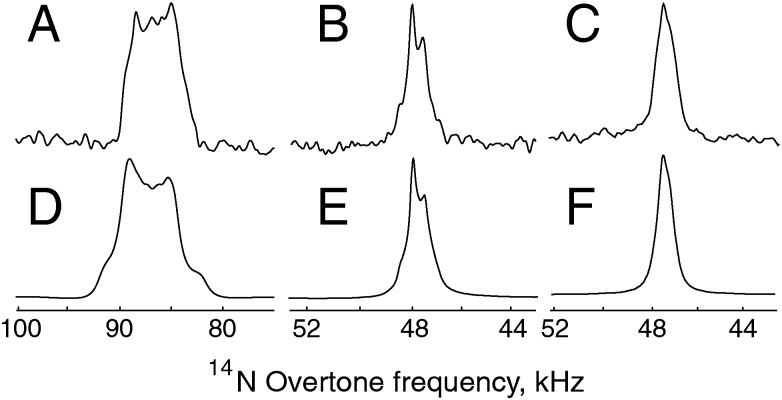
^14^N overtone direct excitation experimental and simulated spectra obtained at the +2 spinning sideband under MAS at 19.84 kHz with a nominal RF amplitude of 55 kHz at 14.1 T and 70 kHz at 20.0 T, as calibrated on a water sample. (A) Experimental data for NAV, 160 000 scans at 14.1 T and with an excitation pulse width *τ*_p_ = 260 μs. (B) Experimental data for glycine, 1024 scans at 14.1 T with *τ*_p_ = 260 μs. (C) Experimental data for glycine, 1024 scans at 20.0 T with *τ*_p_ = 275 μs. (D, E) Simulation for NAV and glycine, respectively, at 14.1 T. (F) Simulation for glycine at 20.0 T. The simulation approach is described in the Theoretical methods section, with the input parameters summarized in Table 1, and probe geometry at the magic angle, without any fitting or adjustment, except for an arbitrary overall scaling multiplier. The simulated spectra used Lorentzian line broadening of 2000 Hz for NAV, 300 Hz for glycine.

The signals obtained through direct excitation of the ^14^N overtone signal of glycine and NAV at 14.1 T and 20.0 T, as well as the corresponding simulations, are shown in Fig. 3. All measurements were performed at *ω*_*r*_/2π = 19.84 kHz. While overtone transitions are insensitive to the first order quadrupolar interaction, lines can still be significantly broadened by higher order terms. However, it is still possible to record direct overtone spectra in a matter of minutes for glycine, which has a line width of approximately 0.83 kHz at 14.1 T (Fig. 3B). NAV is more challenging system because its larger quadrupolar interaction broadens the observed overtone signal to about 5 kHz. Combined with the long dead time of 70 μs, this results in an acquisition time of about 17 hours for the data displayed in Fig. 3A.

As expected, the line shape for glycine is significantly narrower at higher magnetic field (Fig. 3C), but there is no noticeable loss in signal intensity. For all systems, the line shape and peak position can be reproduced by numerical simulations. The simulation conditions and parameters are summarized in Table 1 – the protons are kept in the direct excitation simulation to make sure that identical spin systems are being compared for the two methods. For glycine, we assume that the nitrogen–proton interaction can be replaced by a spin pair with the proton on the symmetry axis to account for the NH_3_ rotation.^61,62^ NAV is also more challenging from the simulation point of view, as convergence requires a large number of orientations in the spherical grid. As a consequence, typical simulation times on a contemporary workstation are up to an hour for direct excitation.

### Spin echo experiments and simulations

The spin echo sequence is shown in Fig. 2(C). In conventional spin echo measurements on spin-1/2 species, the times *τ*_1_ and *τ*_2_ are adjustable delays of similar duration, the 90° and 180° RF pulses provide signal excitation and inversion respectively and are typically strong enough to neglect the internal Hamiltonian during the pulse. The spin echo is an essential step within the PRESTO-II sequence for polarization transfer discussed below, but to date there has been no report of overtone spin-echo spectra. In order to achieve a spin echo on the overtone transition, some modifications of the “traditional” approach are essential, since overtone pulses are so long that there is a significant contribution from the internal Hamiltonian during the pulse (soft pulses). The pulse durations for the optimal echo do not match the durations obtained through direct optimization of the pulse lengths, and overtone pulse durations of 360–720 μs and 170–340 μs for glycine and NAV (in analogy to PRESTO). The excitation pulses are consistently longer than the expected 90 degree pulse duration for both systems.

**Fig. 4:**
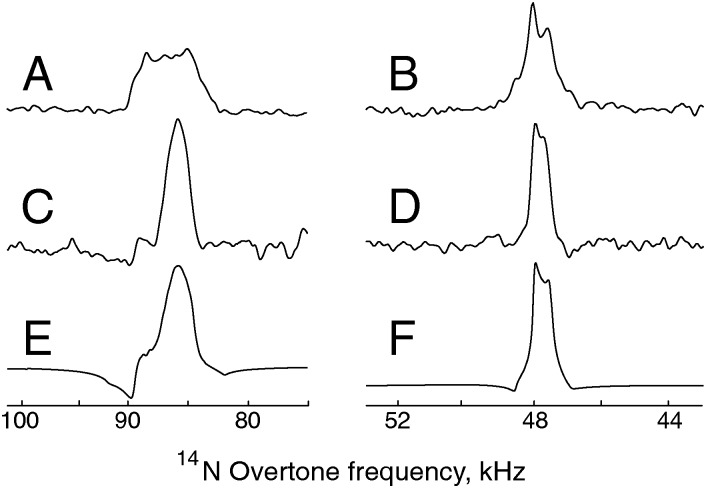
Experimental direct excitation and spin-echo spectra and simulations at 14.1 T and 19.84 kHz on resonance with the +2 spinning sideband of the ^14^N overtone signal. (A) Experimental direct excitation on NAV with 160 000 scans. (B) Experimental direct excitation on glycine with 1024 scans. (C) Experimental spin-echo data on NAV with 40 000 scans and durations of 170 μs and 340 μs for the overtone pulses, *τ*_1_ = 0 μs and *τ*_2_ = 10 μs for the delays. The signal enhancement is 2.0 in terms of peak height, and 0.8 in terms of peak area. (D) Experimental spin-echo spectrum on glycine, 1024 scans and durations of 360 μs and 720 μs for the overtone pulses, *τ*_1_ = 0 μs and *τ*_2_ = 10 μs. The signal enhancement is 1.2 in terms of peak height, and 0.8 in terms of peak area. (E and F) Numerical simulations of spin-echoes in data sets C and D, using the spin system parameters given in Table 1 and the same signal processing parameters as those used for the experimental data.

Optimal signal intensity was achieved under unusual conditions, with no delay between the overtone pulses. Experimental data at 14.1 T and corresponding simulations were obtained for *τ*_1_ = 0, *τ*_2_ = 10 μs and are shown in Fig. 4, together with the corresponding simulations. Data and simulations are presented with no phase correction. More “traditional” echo experiments, with a non-zero delay between the overtone pulses, are provided in the ESI† (see Fig. S2), with only minor signal loss. Spin echo lines are visibly narrower than direct excitation, there is a small loss in integrated signal intensity, but the echo signal height increases by a factor of 1.2 and 2.0 respectively for glycine and NAV, without any need for a change in the pulse delay.

### PRESTO-II ^14^N overtone experimental spectra and simulations

The PRESTO-II sequence has already been used to transfer polarization from high-gamma species to other spin 1/2 nuclei as well as to half-integer quadrupolar spins.^33,34^ The strength of PRESTO is its simplicity when it comes to manipulation of the low-gamma spins, in this case the ^14^N overtone. While conventional CP methods are relatively demanding on both channels, as they typically require a spin lock, PRESTO-II consists of a rather simple spin echo on the *S* channel while the complicated and demanding part of the pulse sequence is on the *I* channel.

**Fig. 5:**
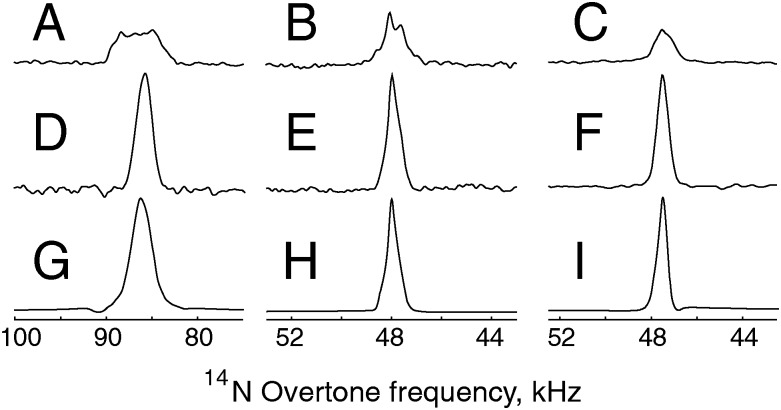
Experimental direct excitation and PRESTO-II spectra and PRESTO-II simulations at 19.84 kHz on resonance with the +2 spinning sideband of the ^14^N overtone signal. (A) Experimental direct excitation on NAV with 160 000 scans at 14.1 T (B) Experimental direct excitation on glycine with 1024 scans at 14.1 T. (C) Experimental direct excitation on glycine with 1024 scans at 20.0 T, *τ*_1_ = 0 μs and *τ*_2_ = 10 μs. (D) Experimental PRESTO-II data on NAV at 14.1 T with 20 000 scans and durations of 170 μs and 340 μs for the overtone pulses. The signal enhancement is 3.8 in terms of peak height, and 2.0 in terms of peak area. (E) Experimental PRESTO-II spectrum on glycine at 14.1 T with 1024 scans and durations of 360 μs and 720 μs for the overtone pulses, *τ*_1_ = 0 μs and *τ*_2_ = 10 μs. The signal enhancement is 2.5 in terms of peak height, and 1.5 in terms of peak area. (F) Experimental PRESTO-II spectrum on glycine at 20.0 T, 1024 scans and durations of 375 μs and 750 μs for the overtone pulses, *τ*_1_ = 43 μs and *τ*_2_ = 40 μs. The signal enhancement is 3.3 in terms of peak height, and 2.0 in terms of peak area. (G–I) Numerical simulations of PRESTO-II, corresponding to data sets D–F, using the spin system parameters given in Table 1 and the same signal processing parameters as those used for the experimental data.

To enhance the sensitivity of the directly detected overtone signal, PRESTO-II was applied to transfer polarization from protons. The resulting spectra for glycine and NAV are shown in Fig. 5 for the symmetry *R*1852 at *ω*_*r*_/2π = 19.84 kHz and two fields (PRESTO-II data were also obtained on glycine at 9.92 kHz and 14.1 T using *R*1871, with similar performance). A comparison between the ^14^N overtone signals as obtained through direct excitation and PRESTO-II, and the corresponding simulations are shown in Fig. 5. There is no enhancement if the signal is measured per unit time using the fully relaxed proton bath as starting condition, as the repetition delay for PRESTO-II is set to 2.5 s, significantly longer than the values used for the ^14^N overtone measurements. In the PRESTO-II experiments reported here we focus purely on the polarization transfer step from protons in order to address strengths and limitations of this approach, hence we implemented a two-step phase cycling which removes the signal coming from direct excitation through the overtone spin echo within the PRESTO sequence. A comparison between PRESTO and spin echo data acquired with a small delay between overtone pulses at 14.1 T is given in the ESI† (Fig. S2). The directly excited spin echo overtone signal could potentially be retained and lead to a further increase of the PRESTO signal intensity and peak shape. PRESTO data acquisition can be achieved much more time-effectively by pre-saturating the ^1^H bath and transferring polarization with just 1 s delay, while retaining about 80% of the signal (see Fig. S3 in ESI†).

When comparing experimental signal intensity for a given number of scans, the PRESTO-II experiment leads to signal enhancement by a factor of 2.5 and 3.8 for glycine and NAV respectively at 14.1 T, and by a factor of 3.3 on glycine at 20.0 T. PRESTO-II gives a narrower signal than direct excitation; hence the signal enhancement is smaller in terms of peak area, with factors of 1.5 and 2.0 respectively when compared to direct excitation on glycine and NAV at 14.1 T, and 2.0 for glycine at 20.0 T. The simulated PRESTO-II overtone peak position and line shape are in good agreement with the experimental data for both samples, and are quite sensitive to the size of the quadrupolar interaction. The signal enhancement with PRESTO is only moderately affected by an increase in magnetic field, which is encouraging for applications to more complex systems, where the added resolution and line narrowing from higher magnetic fields may be important.

There have been reports of polarization transfer to the overtone transitions under MAS using ramped CP, but with unclear evidence of signal enhancement due to the ^1^H to ^14^N overtone transfer. Rossini and co-workers have reported significant signal enhancements using DNP,^30^ but these enhancements come mainly from the hyperpolarization of the ^1^H bath through DNP rather than the polarization transfer,^30^ enhancements we expect would be mirrored when applying PRESTO. Transfer from protons is an essential step when using DNP methods and the high-gamma species allow for a more effective spread of the hyperpolarization signal, hence direct excitation of ^14^N under those conditions would be much more unfavourable, despite its shorter relaxation time.

The experimental enhancement in the signal intensity is moderate on glycine, but is significant on NAV. This is encouraging, as it indicates the potential for applications in biological samples, because NAV is a good model system for the peptide bond. In the past, enhancement factors close to 6 have been reported for static solids, in particular for single crystals, using static adiabatic CP methods.^27^ PRESTO-II achieves significant enhancement in peak heights, but with the advantages of MAS in terms of partial averaging of other anisotropic interactions. The combination of higher signal enhancement provided by polarization transfer in combination with the narrowing of the overtone transition with PRESTO-II may prove advantageous in more complex systems, but only if narrowing of the lines can be maintained while enhancing the very modest bandwidth of the current PRESTO-II sequence (see Fig. S4 in ESI†). The reduced bandwidth of PRESTO is attributed to the reduced bandwidth of the spin-echo part of the sequence, which we will address in future with the use of broadband instead of continuous-wave pulses.

This approach complements methods for indirect overtone detection *via* protons under ultra-fast MAS. PRESTO-II operates at moderate spinning frequencies and RF fields easily achievable on common 3.2 mm or 4 mm probes. On the other hand, methods based on detection *via* ^1^H have been very successfully demonstrated on a very fast spinning probes,^31,32^ but achieving optimal performance in that case requires a combination of both high RF power and high spinning speed.

### Orientation dependence of direct excitation and PRESTO signals

The origin of the line narrowing observed on all samples with spin echoes and PRESTO-II has been further investigated, as it was quite surprising. Despite the same timings as for the spin echoes (Fig. 4), the PRESTO-II signals are moderately narrower. We have analyzed the position and intensity of the signals excited by each orientation of our *γ*-averaged (due to automatic spinner phase averaging within Floquet formalism^49^) {*α*,*β*} Lebedev powder grid to establish more clearly which orientations are preferentially excited by direct excitation and by PRESTO.

**Fig. 6:**
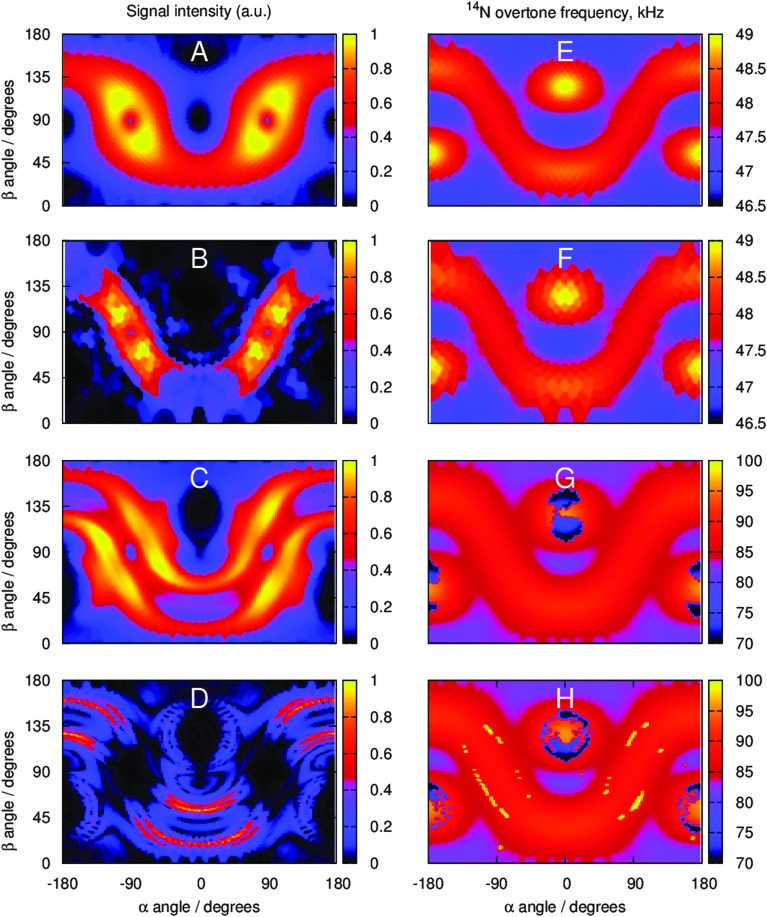
Simulated signal intensity (A–D) and position (E–H) distributions with respect to {*α*,*β*} angles of the spherical averaging grid (two-angle Lebedev sets specified in Table 1, *γ* angle is averaged over by the Floquet formalism). (A) Signal intensity distribution for direct excitation on glycine. (B) Signal intensity distribution for PRESTO-II acquisition on glycine. (C) Signal intensity distribution for direct excitation on NAV. (D) Signal intensity distribution for PRESTO-II acquisition on NAV. **(**E) Signal position distribution for direct excitation on glycine. (F) Signal position distribution for PRESTO-II acquisition on glycine. (G) Signal position distribution for direct excitation on NAV. (H) Signal position distribution for PRESTO-II acquisition on NAV.

Simulation results using the parameters in Table 1 are shown in Fig. 6. While both direct excitation and PRESTO excite the same spectral regions, signal intensity distribution is significantly different and there are many more orientations which do not contribute much to the overall signal intensity when using PRESTO. The jagged intensity distribution pattern obtained for NAV also explains why a large number of spherical grid angles is required to achieve convergence. The narrow line shapes seen in PRESTO spectra are therefore the result of the very long overtone transition pulses only exciting a part of the full powder pattern.

### Two-dimensional ^1^H–^14^N overtone correlation *via* PRESTO-II

**Fig. 7:**
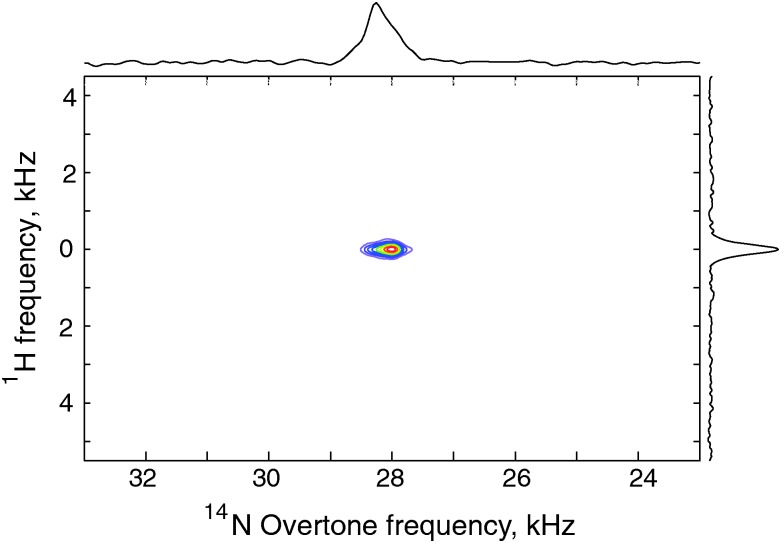
^1^H–^14^N overtone correlation experiment obtained with pulse sequence in Fig. 2B. The 2D data set consists of 96 *t*_1_ increments, 48 scans per increment using the PRESTO-II sequence with symmetry *R*1871 at *ω*_*r*_/2π = 9.92 kHz.

The development of an efficient polarization transfer method at moderate spinning speeds (10–20 kHz) and RF amplitudes allows for the acquisition of multidimensional data sets that would improve signal separation in more complex systems. The PRESTO sequence described above is easily expanded to allow the acquisition of a ^1^H–^14^N overtone correlation spectrum under MAS, as shown in Fig. 7, and obtained with the pulse sequence sketched in Fig. 2B. The application of FSLG homonuclear decoupling during the indirect dimension results in a single correlation, between the nitrogen site and the amine protons.^27,30^

## Conclusions

This work demonstrates that the ^14^N overtone signal can be enhanced using symmetry based sequences like PRESTO-II and those polarization transfer schemes can be employed to perform correlation spectroscopy. The experimental data are consistent with numerical simulations obtained using an algorithm based on frequency-domain detection with an effective Hamiltonian treatment applied on top of Floquet or Fokker–Planck methods. The approach we describe here is faster than the brute-force lab frame simulation; it will help in both the development of improved experimental techniques and in the interpretation of the resulting spectra.

A number of ways are known to enhance the sensitivity of ^14^N overtone detection – both fast MAS and very strong RF fields have been demonstrated to be beneficial for the efficient excitation of the overtone transition.^31,32^ Here we have investigated an alternative approach to ^14^N overtone signal enhancement that relies on the efficient transfer of polarization from the abundant protons to the ^14^N overtone using the PRESTO-II sequence. We found this method to be effective with moderate RF fields and MAS spinning frequencies. This work also demonstrates alternative methods for obtaining multidimensional correlation experiments involving ^14^N overtone and describes the computational tools required to continue their development.
