# Supplementary material for: 14N overtone NMR under MAS: signal enhancement using symmetry-based sequences and novel simulation strategies
Source: Phys Chem Chem Phys. 2015 Feb 9;17(9):6577–87. doi: 10.1039/c4cp03994g (PMC4673505; doi:10.1039/c4cp03994g)
Supplement: Supplementary file 1 [file CP-017-C4CP03994G-s001.pdf]

Electronic Supplementary Material (ESI) for Physical Chemistry Chemical Physics.
This journal is © the Owner Societies 2015

Supporting information for

"^{14}N Overtone NMR under MAS: signal enhancement using symmetry-based sequences and novel simulation strategies "

Ibraheem Haies[a,b], James A. Jarvis[c], Harry Bentley[a], Ivo Heinmaa[d], Ilya Kuprov[a], Philip T.F. Williamson[c], Marina Carravetta[a]*.

[a] *School of Chemistry, University of Southampton, SO17 1BJ, Southampton, United Kingdom.*

[b] *Department of Chemistry, College of Science, University of Mosul, Mosul, Iraq.*

[c] *School of Biological Sciences, University of Southampton, SO17 1BJ, Southampton, United Kingdom.*

[d] *National Institute of Chemical Physics and Biophysics Akadeemia tee 23, Tallinn, 12618, Estonia.*

(Dated: 11 September 2014)

FIGURE S1:

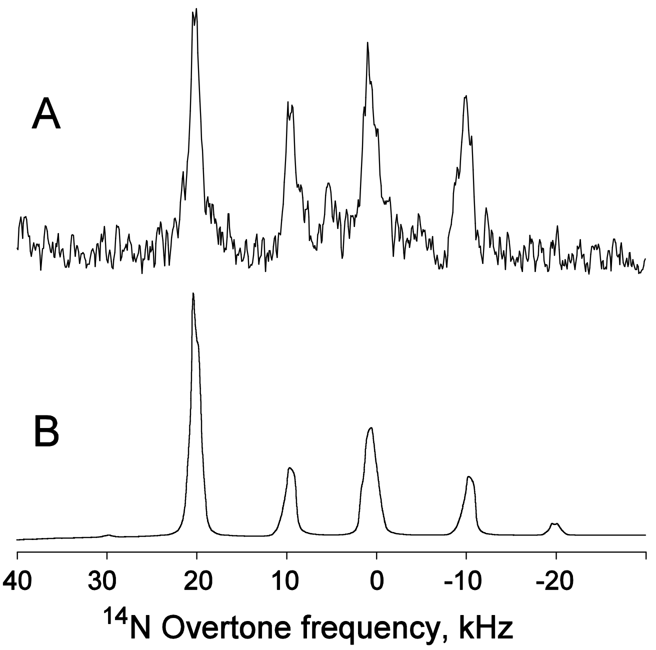

Fig. S1. (A) Experimental overtone sidebands, acquired with the RF carrier set on resonance with the central overtone transition, at $\omega_r/2\pi$ = 9.92 kHz, using 450000 scans and a pulse width of 50 μs. The first 109 μs from the end of the RF pulse were removed (dead-time and left-shift) before the FID was Fourier transformed. The resulting spectra are reported in magnitude mode. (B) Simulation, using the formalism described in the main text with GSQ rank 29 and Floquet rank 10, of the same experiment using experimental probe geometry and pulse amplitudes.

The sideband spectrum was acquired on resonance for the overtone center band. As can be seen from the above spectra, the intensity of the full sideband family is much weaker (data acquired with 450,000 scans, with 38mg of glycine in a 3.2mm thin walled rotor) than the detection of the single sideband (see main manuscript). The removal of the first 100μs of signal acquisition due to severe ringing was also reported by O'Dell and Brinkmann. This large time gap causes significant phase distortions, and thus data is presented in magnitude mode and compared with simulations processed in an identical fashion. The experimental data in Fig. S1A and simulations in Fig. S1B show that the $\pm\omega_r$ spinning sidebands, $+2\omega_r$spinning sideband, and the center band peaks can be clearly detected. The $-2\omega_r$ spinning sideband is expected to be very weak and did not appear at these conditions. The $+2\omega_r$ spinning sideband is the most intense peak and used in all studies within the paper.

FIGURE S2:

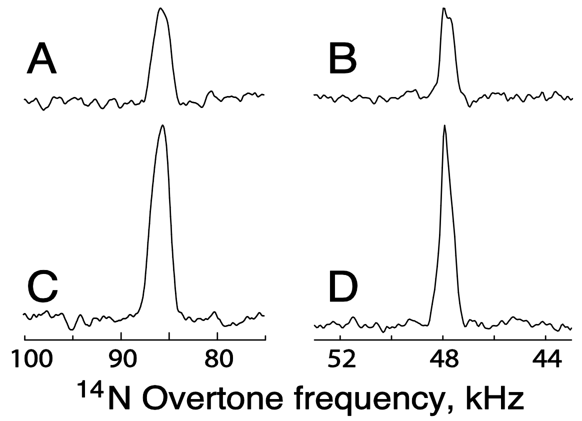

Fig. S2. Experimental comparison between spin-echo and PRESTO-II data at 14.1 T and a spinning frequency of 19.84 kHz on resonance with the +2 spinning sideband of the ^{14}N overtone signal. (**A**) Spin echo on NAV, 40000 scans with $\tau_1 = 116.6\mu$ s, $\tau_2 = 116.4\mu$ s, overtone pulses of 170 and 340 μs. The signal height is 1.9 times larger than direct excitation and the integrated signal intensity changes by a factor of 0.8. (**B**) Spin echo on glycine, 1024 scans with $\tau_1 = 21.6\mu$ s, $\tau_2 = 20.0\mu$ s, overtone pulses of 360 and 720. The signal height is 1.2 times larger than direct excitation and the integrated signal intensity changes by a factor of 0.8. (**C**) PRESTO-II on NAV, 20000 scans with $\tau_1 = 116.6\mu$ s, $\tau_2 = 116.4\mu$ s, overtone pulses of 170 and 340. The signal height is 3.7 times larger than direct excitation and the integrated signal intensity increases by a factor of 1.9. (**D**) PRESTO-II on glycine, 1024 scans with $\tau_1 = 21.6\mu$ s, $\tau_2 = 20.0\mu$ s, overtone pulses of 360 and 720 μs and 2.5 s repetition delay. The signal height is 2.4 times larger than direct excitation and the integrated signal intensity increases by a factor of 1.4.

Besides the case of no delay between overtone pulses, we also provide a more conventional example of overtone spin echo, with a delay between pulses in the range of tens to hundreds of microseconds. The timings were chosen in such a way to be compatible with a PRESTO-II sequence where the second overtone pulse starts exactly at the end of the PRESTO reconversion block. As can be seen, the PRESTO spectra are only moderately narrower than the corresponding spin echo spectra under these conditions.
Efficiencies are nearly identical to what reported in the main paper both for PRESTO and for the echo.

FIGURE S3:

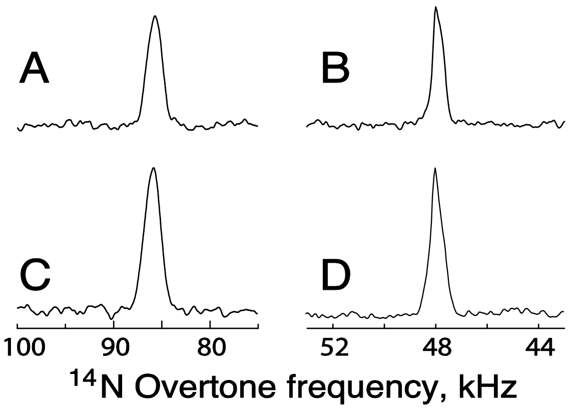

Fig. S3. Experimental comparison between PRESTO-II data at 14.1 T and 19.84 kHz on resonance with the +2 spinning sideband of the ^{14}N overtone signal, acquired using with $\tau_1 = 0\mu$s, $\tau_2 = 10.\mu$s acquired with different repetition delays. (**A**) NAV, 20000 scans with overtone pulses of 170 and 340 μs and 1 s repetition delay. The signal height is 2.4 times larger than direct excitation and the integrated signal intensity increases by a factor of 1.5. (**B**) Glycine, 1024 scans overtone pulses of 360 and 720 and 1 s repetition delay. The signal height is 2.0 times larger than direct excitation and the integrated signal intensity increases by a factor of 1.2. (**C**) NAV, 20000 scans with overtone pulses of 170 and 340 with 2.5 s pulse delay. (**D**) Glycine, 1024 scans with overtone pulses of 360 and 720 μs and 2.5 s repetition delay.

Time-effective PRESTO-data acquisition is possible if we reduce the pulse delay from 2.5s used in the key paper to show the maximum enhancement from a fully relaxed ^1H signal, to only 1 s. This leads to a PRESTO-II sequence in which the signal per unit time is about equal of slightly larger than the signal with direct excitation.

FIGURE S4:

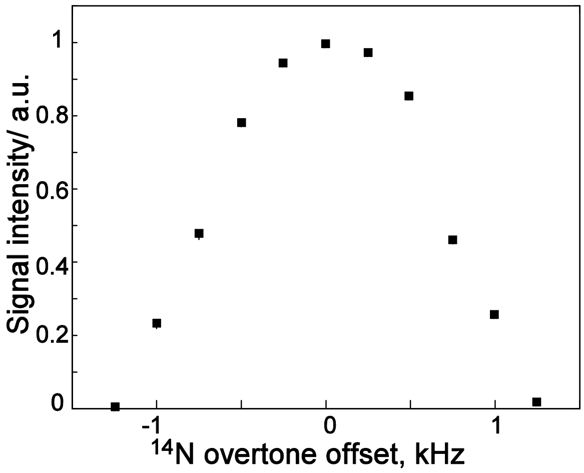

Fig. S4. Experimental PRESTO-II signal intensity for glycine at 14,1 T, 19.84 kHz spinning frequency, 1024 with 55 kHz RF power on the overtone transition as a function of the offset in the ^{14}N overtone field. There is a very strong offset dependence and the overall bandwidth is about 1 kHz.
